# Pharmacist Intervention Models in Drug–Drug Interaction Management in Prescribed Pharmacotherapy

**DOI:** 10.3390/pharmacy13060167

**Published:** 2025-11-17

**Authors:** Ivana Samardžić, Ivana Marinović, Iva Marović, Nikolina Kuča, Vesna Bačić Vrca

**Affiliations:** 1 Department of Clinical Pharmacy, University Hospital Dubrava, 10 000 Zagreb, Croatia; ivana.samardzic1@gmail.com (I.S.);; 2 Mandis-Pharm Pharmacy Zagreb, 10 000 Zagreb, Croatia; 3 Faculty of Pharmacy and Biochemistry, University of Zagreb, 10 000 Zagreb, Croatia

**Keywords:** drug–drug interactions, pharmacist, interventions, pharmacotherapy

## Abstract

Drug–drug interactions (DDIs) are one of the most common problems related to drug administration which represent a risk for patient safety. Considering their position in the healthcare system, pharmacists should be more proactively involved in DDI management. The paper shows representation of DDI intervention models in each DDI category. This research enrolled outpatients prescribed pharmacotherapies from 40 randomly selected community pharmacies. DDIs were analyzed using Lexicomp^®^ Lexi-InteractTM Online (Lexi-Comp, Inc., Hudson, NY, USA) software. Clinical pharmacists’ panel, according to the necessary interventions, determined an independent model of pharmacist interventions (category 1) and models that require cooperation with physicians (category 2) for DDI management. In total, 4107 patients were enrolled in the study. Mean patient age was 67.5; they were mostly women (56.5%) and had on average of 3.4 diagnosis and 5.5 prescription drugs. Overall, 14,175 potential clinically significant DDIs were identified: 83.3% of C, 15.4% of D, and 1.3% of X category. At least one potential DDI was found in 78.6% of patients. Models of pharmacist DDI interventions in collaboration with a physician (category 2) were more prevalent than independent models (category 1): 57.5% vs. 42.5% in C category DDIs, 97.8% vs. 2.2% in D category, and 100% vs. 0% in category X DDIs. This research aimed to gain an insight into the distribution of interventions in DDI management models between physicians and pharmacists, which can contribute to more efficient pharmaceutical care and visibility.

## 1. Introduction

In the modern healthcare system, the role of pharmacists has significantly expanded to comprehensive pharmacotherapy management. Pharmacy interventions are particularly developing in terms of optimizing and rationalizing therapy, increasing medication safety, and improving treatment outcomes [1,2,3]. During the dispensing process, pharmacists can detect various pharmacotherapy problems, which can affect treatment effectiveness and jeopardize patient safety. The most common pharmacotherapy problems related to medication dispensing include unjustified use of the drug, lack of necessary therapy, inappropriate drug, inappropriate dose, drug interactions, side effects, inappropriate drug formulation or route of administration, and non-adherence [4].

Drug–drug interactions (DDIs) are one of the key problems associated with pharmacotherapy optimization. DDIs are defined as the change in the effect of one drug due to the simultaneous or previous administration of another drug [5]. DDIs can complicate the course of treatment, cause or prolong hospitalization, and increase pharmacoeconomic costs [6]. DDIs can be targeted or unintended, in both cases they require optimal management. Another key categorization of DDIs is according to the mechanism of action, which includes pharmacokinetic and pharmacodynamic interactions. Pharmacokinetic interactions occur at the level of absorption, distribution, metabolism, or elimination, and change the drug concentration in the body, while pharmacodynamic interactions occur at the level of receptors or physiological systems and change the effect of the drug without changing its concentration.

Patients with polypharmacy, elderly patients, transplant patients, and patients with impaired liver and kidney function are especially vulnerable to DDIs [7,8,9,10]. The problems related to DDIs in community pharmacy are often underestimated compared to those in hospital settings. In outpatient settings, there is a lack of systematic control of DDIs and related pharmacotherapy problems may remain unrecognized in a timely manner or not at all [11,12]. Patients often have multiple medications recommended by different specialists who usually do not have insight into patients’ complete therapy. Therefore, pharmacotherapy problems can only be recognized at the time of drug dispensing. Models of pharmacist interventions are essential for the development of uniform and standardized pharmaceutical care. This research aimed to gain an insight into the distribution of interventions in DDI management models between physicians and pharmacists, which can contribute to more efficient pharmaceutical care and visibility.

## 2. Materials and Methods

### 2.1. Data Collection

The research was conducted as a retrospective, consecutive analysis of prescribed and dispensed outpatient pharmacotherapy. In total, 4107 pharmacotherapy patients’ cards were analyzed. The pharmacy program records every patient’s visit to the pharmacy and all prescription medications that the patient receives. The pharmacotherapy card is designed as a single document per patient and provides a chronological overview of all medications that have been dispensed to a patient in the pharmacy. The pharmacotherapy card also records the amount of the dispensed drug, the drug form, the dose, the interval, and the recommended route of administration.

Over-the-counter (OTC) drugs were not included in the analysis, since mandatory and systematic records of OTC drugs are not available in community pharmacies in the Republic of Croatia. The research included 40 community pharmacies. Pharmacies were randomized using the Research Randomizer program according to the official register of community pharmacies of Croatian national chamber. An equal number of pharmacies were included from Croatian regions, and from rural and urban areas. Also, an equal number of pharmacotherapy cards were requested from each pharmacy. Criteria for regions, urbanity and rurality are determined according to the Eurostat classification [13]. The analysis included pharmacotherapies of patients over 18 years of age who were prescribed at least two prescription drugs for systemic use. The elderly patients were considered to be patients aged 65 years and older.

### 2.2. Models of Pharmacist Interventions

The panel of clinical pharmacist specialists developed models of interventions for DDIs. The panel of clinical pharmacists had to differentiate independent pharmaceutical interventions from those requiring collaboration with a physician in order to create a final model of DDI management by scope of pharmaceutical care. The model for management of specific potentially clinically significant interactions can consist of a single pharmacist intervention or a combination of pharmacist interventions. After analyzing the necessary interventions for individual DDIs, the following models of pharmacist interventions in DDI management were formed. Types of pharmacist interventions are shown in Table 1. Type A interventions (labeled A1 to A4) are those that the pharmacist can perform independently in accordance with the pharmaceutical care scope, while type B interventions (labeled B1 to B11) are those that the pharmacist recognizes as necessary and recommends to the physician. Type B interventions include interventions that require, for example, monitoring specific clinical parameters, a change in drug or dose, and shortening the period of taking the medication. Physicians can accept or reject recommended interventions after assessing the parameters of the patient’s clinical status and treatment plan. The model of interventions for DDI can constitute type A interventions, type B interventions, or combination of type A and type B interventions. As a result, models are divided into two categories:

Category 1—independent model of pharmacist interventions (includes only one or more type A interventions per DDI)

Category 2—model of pharmacist DDI interventions in collaboration with a physician (includes combinations of type A and type B interventions or only type B interventions per DDI).

### 2.3. DDI Interaction Analysis

DDIs were analyzed for all included patients using the Lexicomp program, which categorizes interactions according to the degree of clinical significance [14].

For DDIs Lexicomp uses the following clinical severity levels:

A—No known interaction

B—No clinical significance

C—Necessary therapy monitoring

D—Consider therapy modification 

X—The combination should be avoided

Pharmacist intervention models were proposed only for clinically significant interactions (C, D, and X levels).

### 2.4. Statistical Analysis

Frequencies and percentages by category were calculated for categorical variables, while for continuous variables, descriptive parameters are presented, including the result of the Shapiro–Wilk test of normality of distribution. Since this is a large sample, the Shapiro–Wilk test is overly sensitive to deviations from normality, and an estimate of the skewness and flatness of the distribution was used to assess the adequacy of the use of parametric procedures. In the displays of descriptive data for normally distributed variables, the arithmetic mean, standard deviation, and total range are shown, while for a variable whose distribution deviates from normal, the median, first and third quartiles, and total range are reported.

A multivariate logistic regression analysis was performed to determine the association of the occurrence of DDIs with certain risk factors, including gender, age, comorbidities, and number of prescribed medications. Pearson’s correlation coefficient was used to analyze the correlation between drug interactions with gender and polypharmacy. *p* = 0.05 or less was considered statistically significant.

## 3. Results

A total of 4107 patient pharmacotherapy cards were included in the study. The patients’ characteristics according to age are presented in Table 2. The average age of patients was 67.5 years (range 18–102). In all, 62.8% of patients were aged 65 years or older. The representation of women in the sample was higher (56.5%). In total, 22,668 prescribed medications were identified. Patients had an average of 5.5 prescribed medications (range 2–18). Overall, 55.5% of patients were prescribed 5–10 medications, and 4.4% of patients were prescribed more than 11 medications.

In total, 14,026 diagnoses were identified. The average number of diagnoses according to the International Classification of Diseases (ICD) was 3.4 (range 1–11). Most patients had more than one diagnosis, 50.3% had 2–3 diagnoses, and 36.9% of patients had 4–6 diagnoses. Only one diagnosis was found in 8% of patients.

There were statistically significantly more elderly patients than those younger than 65 years. A larger proportion of the elderly population were women. Patients older than 65 years had more diagnoses, more drugs, and a significantly higher total number of DDIs and category C interactions. Those younger than 65 years had more X drug interactions, while no significant difference was found in the incidence of D interactions between patients older than 65 years compared to those younger than 65 years.

The patients’ characteristics are presented in Table 3 according to the degree of urbanization. Patients from rural areas were reported to have a statistically significant higher number of medications and more interactions at all levels (C, D, and X). There were no other differences between patients according to the degree of urbanization.

**Figure 1:**
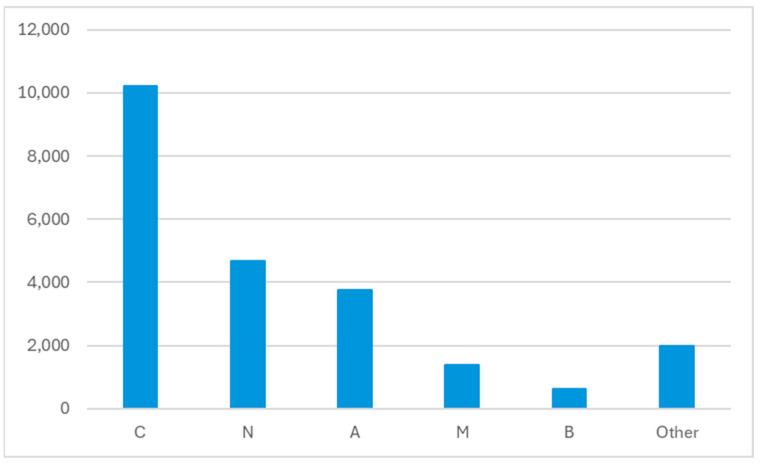
Drugs most represented in prescribed pharmacotherapy according to ATC classification. A—alimentary tract and metabolism; B—blood and blood forming organs; C—cardiovascular system; M—musculo-skeletal system; N—nervous system.

The most frequently prescribed drug groups according to the Anatomical Therapeutic Chemical (ATC) Classification were groups C—cardiovascular system, N—nervous system, and A—alimentary tract and metabolism (Figure 1). The most frequently prescribed medications were amlodipine, bisoprolol, and perindopril (Table 4). The 20 most prescribed medications account for 35% of all prescribed medications.

The analysis of pharmacotherapy has determined 14,175 DDIs (Table 5). Of these, the most common were interactions of level C (83.3%), followed by D (15.4%), while the least common were interactions of level X (1.3%). The largest proportion of patients (57.5%) had 1–5 interactions identified in the prescribed pharmacotherapy. More than 20 potentially clinically significant DDIs were identified in 0.7% of patients. The number of medications was the most significant predictor of a higher number of DDIs, followed by diagnosis and male gender.

Overall, 1907 different pairs of C interactions were identified. The most common interactions included antihypertensive agents. There were 384 different drug pairs in D interactions. The most frequent D interaction was the interaction between diazepam and tramadol. The most common X interaction was between diazepam and olanzapine, while overall, the most common interactions were between NSAIDs. There were 75 different X interaction pairs, of which 45 pairs occurred only once.

**Figure 2:**
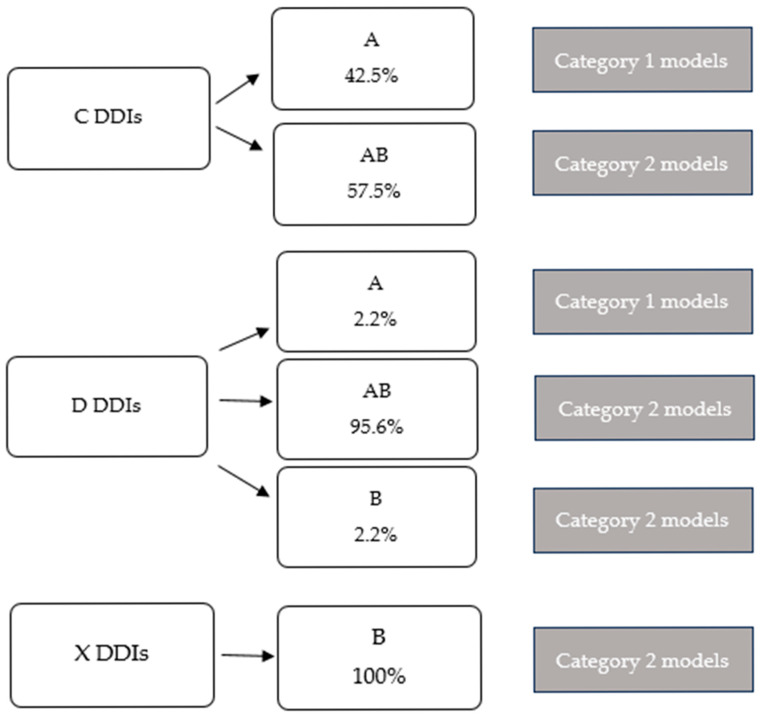
Types and representation of intervention models in the most frequent C (which occurred in more than 50 patients), D (which occurred in more than ten patients), and all X drug–drug interactions.

Types and representation of intervention models according to the level of clinical significance of DDIs are presented in Figure 2. Independent model of pharmacist interventions (category 1 model) includes only type A of interventions, while combinations of A and B interventions or only B interventions represent dependent models that require collaboration with a physician (category 2 model). Clinical pharmacists’ panel determined category 1 and category 2 models of pharmacist interventions for C-level interactions. For D-level interactions, category 1 and category 2 models were also determined. Only category 2 models have been identified for level-X interactions. Models of pharmacist DDI interventions in collaboration with a physician (category 2) were more prevalent across all DDI levels than independent models (category I): 57.5% vs. 42.5% in category C DDIs, 97.8% vs. 2.2% in category D, and 100% vs. 0% in category X DDIs.

Models of pharmacist interventions according to the level of clinical significance for the most frequently identified DDIs are presented in Table 6, Table 7 and Table 8. The most common model for C interactions was A1 + A4 + B1 + B2. The most common model for D interactions was A1 + B1 + B2 + B6. The most common model for X interactions is B3, which indicates a suggestion to the physician to replace one of the drugs.

## 4. Discussion

This nationwide study indicates the high prevalence of potential clinically significant DDIs in prescribed outpatient pharmacotherapy and imposes the need for their management. The high prevalence of interactions also indicates a need for systematic assessment and documentation of pharmacy interventions.

Although DDIs are one of the most frequently cited causes related to patient safety, effective DDI prevention still represents a major challenge for the healthcare system. In our study, at least one potentially clinically significant DDI was identified in 78.6% of patients. Other studies also indicate a significant prevalence of potentially clinically significant interactions in outpatient settings [15,16]. In addition to DDIs incidence, this work also proposed DDI models of pharmacist interventions that can contribute to more efficient pharmaceutical care and visibility. Not managing DDIs can jeopardize effectiveness and safety of pharmacotherapy which can especially affect elderly patients and patients taking multiple medications [7,17,18,19].

In our study, patients had an average of 5.5 medications and more than half of them (55.5%) had 5–10 prescribed medications. In 4.4% of patients, we found more than 11 prescribed drugs. The most commonly used drugs were drugs from group C according to the ATC classification, which is consistent with the annual report on drug utilization by the Croatian Agency for Medicinal Products and Medical Devices [20]. Polypharmacy makes medication management more difficult and significantly increases the risk of DDIs occurring. In general, the prevalence of clinically relevant drug interactions is about 50% in patients taking five medications and almost 100% in those taking ten medications [21]. Polypharmacy is also associated with adverse outcomes including mortality risk, falls, adverse drug reactions, increased length of hospital stay, and readmissions shortly after discharge [22,23,24].

In our study, elderly patients had a statistically significantly higher number of diagnoses and medications, which resulted in a greater number of potential DDIs. Older patients are at high risk of actual DDIs due to decreased physiological functions. Aging decreases liver and kidney function, gastric acid secretion, and albumin levels but there are also changes in the receptor number and sensitivity to which drugs bind [23,25]. Therefore, aging increases the risk of both pharmacokinetic and pharmacodynamic interactions. Older people make up a significant and growing proportion of the population in Europe. In 2024, the elderly (aged 65 and over) made up an estimated 21.6% of the EU population [13]. Demographic changes have important implications for healthcare and drug therapy. This trend is a challenge for the healthcare system and indicates the need for reorganization and creating specially designed interventions for the geriatric population [26,27]. Study findings also highlight the need for tailored approaches in providing pharmaceutical care in rural regions. More drugs and DDIs were observed in patients in rural areas, likely due to limited access to health services and fewer opportunities for pharmacotherapy monitoring and evaluation.

The most common C DDIs involved antihypertensives. Some of these interactions are targeted but also require management to avoid harmful consequences. High representation of PIMs was observed in the most common D interactions. Other research also indicates a significant prevalence of PIMs in determined DDIs in elderly patients [28]. Furthermore, administration of PIMs upon discharge poses a risk for hospital readmission [29]. There are various tools for evaluating PIMs in older patients [30,31]. The study published in 2021 which determined PIMs using the EU(7)-PIM tool revealed the significant presence of PIMs at all levels of clinically relevant DDIs [28]. The highest share of PIMs was in D interactions and the most frequently involved PIM in DDIs was tramadol. This study included hospitalized patients at discharge, while the current study included outpatients [28]. Applying the criteria for PIMs in deprescribing elderly patients can significantly reduce the incidence of D interactions. However, sometimes D interactions are unavoidable, but appropriate interventions should be applied to increase patient safety and minimize the risk of actual DDIs. In our study, X interactions most often involved drugs from group N according to the ATC classification and nonsteroidal anti-inflammatory drugs (NSAIDs). Determined X interactions could result in an increased risk of CNS depression, gastrointestinal toxicity, and QT prolongation. The most frequent determined interactions were mainly pharmacodynamic interactions according to categorization by the mechanism of action.

This research aimed to gain an insight into the distribution of interventions in DDI management models between physicians and pharmacists, which can contribute to more efficient pharmaceutical care and visibility. The clear distinction of responsibilities between physicians and pharmacists in managing DDIs is a significant challenge for clinical practice. Unclear distinctions can lead to delays in intervention, overlapping, or complete lack of intervention. Introducing clear protocols and guidelines is a key to developing a safe and effective DDI management system. The analysis of the necessary interventions was based on the DDI management recommendation database. Lexicomp was chosen for analysis because it is a commonly used tool, has high sensitivity (98%), and specificity (90%) [14]. The panel of clinical pharmacists had to distinguish independent pharmaceutical interventions from dependent ones to create the final DDI model management. Evaluation considered the responsibilities that are in the scope of pharmaceutical care. Although created pharmacy interventions were based on and guided by the existing Lexicomp classification and recommendations, an additional scientific contribution of our work is in identifying the most common drug interactions in the general population and creating a clear and defined workflow for their management and collaboration between physicians and pharmacists.

The highest proportion of independent interventions was determined in DDIs of clinical significance level C, where interventions such as patient counseling, blood pressure monitoring, dosing interval separation, or monitoring of side effects were required. In D interactions, there are fewer independent pharmacist interventions, since such interactions already require a change in medication with a lower risk of interference, dose adjustment, and limitation of drug administration time which is not feasible without cooperation with a physician. The complete absence of independent pharmaceutical interventions was found in X interactions. The pharmacist is often able to identify all potentially clinically significant interactions because of the insight into patient’s entire therapy, including drugs from different specialists and OTC drugs. In addition to recognizing interactions, it is also important that pharmacists provide recommendations for resolving or monitoring potential clinically significant DDIs. A well-developed DDI management protocol accelerates the system, increases its efficiency, and enables standardization. Standardization of pharmacy interventions further enables consistency in practice, improvement in quality of care, quantification, and better visibility.

The implementation of the pharmacist model interventions in clinical practice requires continuity and the development of user programs that will enable precise communication between physicians and pharmacists. The models require regular evaluation so that their application can be continuous and aligned with the latest recommendations and guidelines. The models can change over time according to changes in drug use recommendations, for example, changes in SmPC and new pharmacovigilance information [14]. The implementation of pharmacist model interventions in pharmaceutical care might bring significant cost savings, raise the quality of healthcare, and increase patient safety.

It is difficult to apply models immediately to all possible DDIs, but priority should be given to those DDIs that are most common in the population, those that are most clinically significant, and those that are more severe for a particularly sensitive part of the population, such as the elderly or chronically ill [32]. Further research and analysis are needed to assess the economic, technical, and clinical implications for their implementation and sustainability.

A limitation of this study is the omission of OTC medications from the analyses. OTC medication registers were not available and are not mandatory in community pharmacies. Certain medications identified in X interactions, such as some NSAIDs, are also available as OTC medications. OTC drugs are mostly used as a first-line or initial therapy for a variety of minor diseases or conditions, such as allergies, musculoskeletal discomfort, headaches, heartburn, and cold. However, the safety and effectiveness of any medication with OTC status should be considered as a potential interacting agent when introduced into therapy simultaneously with other prescription medications. Therefore, it would be useful that future prospective research includes OTC drugs in the analysis.

## 5. Conclusions

The research provides insight into the distribution of independent and dependent models of pharmacist interventions in DDI management. An undefined workflow in DDI management can lead to inappropriate intervention or no intervention at all. Models of pharmacist DDI interventions in collaboration with a physician were more prevalent than independent pharmacist models, especially in the DDI categories of higher level of clinical significance. However, in overall DDI management, pharmacists can accelerate the DDI management process and make it more efficient by recognizing a potential interaction, proposing solutions, and providing expert recommendations.

## Figures and Tables

**Table 1:** Overview of independent pharmacy interventions (type A) and dependent pharmacist interventions that require management collaboration with physicians (type B).

Type A—Independent Pharmacist Interventions
A1—Patient counseling and education
A2—Monitoring therapeutic parameters
A3—Change/split dosing interval
A4—Monitoring side effects
Type B—Dependent pharmacist interventions (recommendations to the physician)
B1—Monitoring (therapeutic effect, drug concentration, side effects)
B2—Dose adjustment
B3—Substitution of one of the interacting drugs
B4—Therapy duplication—discontinuation of one of the drugs
B5—Evaluation of drug use
B6—Limiting the duration of drug use
B7—Necessity to introduce an additional drug
B8—Change in pharmaceutical form of the drug
B9—Temporary discontinuation of one interacting drug
B10—Discontinuation of a specific drug with a washout period before introducing a new one
B11—Gradual drug withdrawal

**Table 2:** Patients’ characteristics according to age.

	Total	<65 Years	≥65 Years	*p*
Participants, N (%)	4107 (100)	1528 (47.2)	2579 (62.8)	<0.001
Female gender, N (%)	2322 (56.5)	824 (53.9)	1498 (58.1)	0.010
ICD diagnoses, N mean ± SD range	14,026 3.42 ± 1.65 (1–11)	4636 2.03 ± 1.54 (1–11)	9390 3.64 ± 1.67 (1–11)	<0.001
Medications, N mean ± SD range	22,668 5.52 ± 2.58 (2–18)	7533 4.93 ± 2.45 (2–17)	15,135 5.87 ± 2.59 (2–18)	<0.001
DDIs, N (median) average rang range	14,175 2 (1–5) (0–31)	4817 (2.0) 1926.5 (0–25)	9358 (2.0) 2129.5 (0–31)	<0.001
C interactions, N (median) average rang range	11,803 2 (0–4) (0–28)	3891 (1.0) 1906.9 (0–20)	7912 (2.0) 2141.1 (0–28)	<0.001
D interactions, N (median) average rang range	2182 0 (0–1) (0–13)	835 (0.0) 2034.7 (0–10)	1347 (0.0) 2065.4 (0–13)	0.326
X interactions, N (median) average rang range	190 0 (0–0) (0–3)	91 (0.0) 2079.7 (0–2)	99 (0.0) 2038.8 (0–3)	0.002

ICD—International Classification of Diseases; SD–standard deviation; DDI—drug–drug interaction.

**Table 3:** Patients’ characteristics according to the degree of urbanization.

	Total	Rural Areas	Urban Areas	*p*
Participants, N (%)	4107 (100)	2050 (49.9)	2057 (50.1)	0.913
Age, years mean ±SD range	67.47 ± 13.674 (18–102)	67.35 ±13.617 (18–96)	67.60 ± 13.733 (19–102)	0.566
≥65 years, N (%)	2579 (62.8)	1261 (61.5)	1318 (64.1)	0.093
Female gender, N (%)	2322 (56.5)	1141 (55.7)	1181 (57.4)	0.257
ICD diagnoses, N mean ± SD range	14,026 3.42 ± 1.647 (1–11)	7095 3.46 ± 1.692 (1–11)	6931 3.37 ± 1.599 (1–11)	0.075
Medications, N mean ± SD range	22,668 5.52 ± 2.585 (2–18)	11,545 5.63 ± 2.642 (2–18)	11,123 5.41 ± 2.521 (2–16)	0.005
DDIs, N (median) average rang range	14,175 2 (1–5) (0–31)	7555 2 (1–5) (0–31)	6620 2 (1–4) (0–29)	<0.001
C interactions, N (median) average rang range	11,803 2 (0–4) (0–28)	6261 2 (1–4) (0–24)	5542 2 (0–4) (0–28)	<0.001
D interactions, N (median) average rang range	2182 0 (0–1) (0–13)	1179 0 (0–1) (0–13)	1003 0 (0–1) (0–12)	0.003
X interactions, N (median) average rang range	190 0 (0–0) (0–3)	115 0 (0–0) (0–3)	75 0 (0–0) (0–3)	0.009

ICD—International Classification of Diseases; SD—standard deviation; DDI—drug–drug interaction.

**Table 4:** The most frequently prescribed medications.

ATC	Drug	N
C08CA01	amlodipine	1055
C07AB07	bisoprolol	1038
C09AA04	perindopril	778
A02BC02	pantoprazole	774
N05BA01	diazepam	741
C10AA05	atorvastatin	732
C09AA05	ramipril	718
C03AA03	hydrochlorothiazide	697
A10BA02	metformin	676
C03BA11	indapamide	625
N02AX02	tramadol	588
C03CA01	furosemide	544
N05BA12	alprazolam	514
M01AE01	ibuprofen	488
N02BE01	paracetamol	468
H03AA01	levothyroxine	426
C09CA03	valsartan	370
C10AA07	rosuvastatin	356
C07AB12	nebivolol	315
G04CA02	tamsulosin	304

**Table 5:** Potential clinically significant DDIs in prescribed pharmacotherapy.

	Total	DDIs Categories		
		C	D	X
DDIs, N (%)	14,175 (100)	11,803 (83.3)	2182 (15.4)	190 (1.3)
Patients with DDIs, N (%)	3228 (78.6)	3043 (74.1)	1289 (31.4)	169 (4.1)
DDIs, median (IQR) (range)	2 (1–5) (0–31)	2 (0–4) (0–28)	0 (0–1) (0–13)	0 (0–0) (0–3)

DDIs—drug–drug interactions; IQR—interquartile range.

**Table 6:** Models of pharmacist interventions for the most frequently identified DDIs of level C.

C DDIs			
Drug	Drug	Potential DDI Consequence	DDI Management and Model
perindopril	indapamide	Indapamide may increase the nephrotoxic and hypotensive effects of ACEIs.	Counsel the patient Monitor blood pressure Monitor renal function Adjust dose A1 + A4 + B1 + B2
valsartan	HCTZ	Hydrochlorothiazide may increase the hypotensive effect of valsartan. Valsartan may increase the serum concentration of hydrochlorothiazide.	Counsel the patient Monitor blood pressure Monitor renal function and electrolytes A1 + A4 + B1
lizinopril	HCTZ	Hydrochlorothiazide may enhance the nephrotoxic and hypotensive effects of ACEIs.	Counsel the patient Monitor blood pressure Monitor renal function Adjust dose A1 + A4 + B1 + B2
ramiprile	HCTZ	Hydrochlorothiazide may enhance the nephrotoxic and hypotensive effects of ACEIs.	Counsel the patient Monitor blood pressure Monitor renal function Adjust dose A1 + A4 + B1 + B2
metformin	perindopril	ACEIs may increase the risk of side effects/toxic effects of metformin.	Counsel the patient Increase monitoring for hypoglycemia signs Monitor for risk/signs of lactic acidosis A1 + A4 + B1

DDI—drug–drug interaction; HCTZ—hydrochlorothiazide; ACEI—angiotensin converting enzyme inhibitor.

**Table 7:** Models of pharmacist interventions for the most frequently identified DDIs of level D.

D DDIs			
Drug	Drug	Potential DDI Effect	DDI Management and Model
diazepam	tramadol	Increased risk of CNS depression.	Counsel the patient Increase patient monitoring Limit drug doses Limit the duration of drug use A1 + B1 + B2 + B6
alprazolam	tramadol	Increased risk of CNS depression.	Counsel the patient Increase patient monitoring Limit drug doses Limit the duration of drug use A1 + B1 + B2 + B6
diazepam	zolpidem	Increased risk of CNS depression.	Counsel the patient Increase patient monitoring Adjust medication doses Evaluate therapy A1 + B1 + B2 + B5
bisoprolol	moxonidine	Alpha2-agonists may potentiate the AV-blocking effect of beta-blockers. Sinus node dysfunction may also be potentiated. Beta-blockers may potentiate the rebound hypertensive effect of alpha2-agonists. This effect may occur when the alpha2-agonist is abruptly withdrawn.	Counsel the patient Increase patient monitoring Consider modification of therapy Gradually withdraw the drug A1 + B1 + B5 + B11
alprazolam	zolpidem	Increased risk of CNS depression.	Counsel the patient Adjust medication doses Limit drug doses Evaluate therapy A1 + B1 + B2 + B5

DDI—drug–drug interaction; CNS—central nervous system.

**Table 8:** Models of pharmacist interventions for the most frequently identified DDIs of level X.

X DDIs			
Drug	Drug	Potential DDI Effect	DDI Management and Model
diazepam	olanzapine	Olanzapine may enhance the effects of benzodiazepines.	Avoid concomitant use, consider another drug combination. B3
furosemide	promazine	Loop diuretics may increase the QTc potential of promazine.	Avoid concomitant use, consider another drug combination. B3
alprazolam	olanzapine	Olanzapine may enhance the effects of benzodiazepines.	Avoid concomitant use, consider another drug combination. B3
carbamazepine	tramadol	Tramadol may increase the CNS depressant effect of carbamazepine. Tramadol may decrease the therapeutic effect of carbamazepine. Carbamazepine may decrease the concentration of tramadol.	Avoid concomitant use, consider another drug combination. B3
diclofenac	ibuprofen	Increased risk of gastrointestinal toxicity.	Duplication of medications, it is necessary to exclude one of the medications from the same therapeutic group. B4

DDI—drug–drug interaction; CNS—central nervous system.

## Data Availability

The original contributions presented in this study are included in the article. Further inquiries can be directed to the first author.

## References

[1] Svensson A. (2024). The Integral Role of Pharmacists in Patient Centered Medication Management. J. Basic Clin. Pharma..

[2] Zellars M. (2024). The evolving role of clinical and hospital pharmacists in patient-centered care. Asian J. Biomed. Pharm. Sci..

[3] Farhat N., Abbas M. (2025). Planning and Developing a Clinical Pharmacy Practice. StatPearls.

[4] Schindler E., Richling I., Rose O. (2021). Pharmaceutical Care Network Europe (PCNE) drug-related problem classification version 9.00: German translation and validation. Int. J. Clin. Pharm..

[5] Juurlink D.N., Mamdani M., Kopp A., Laupacis A., Redelmeier D.A. (2003). Drug–drug interactions among elderly patients hospitalized for drug toxicity. JAMA.

[6] Arnold R.J.G., Tang J., Schrecker J., Hild C. (2018). Impact of Definitive Drug–Drug Interaction Testing on Medication Management and Patient Care. Drugs Real World Outcomes.

[7] Palleria C., Di Paolo A., Giofrè C., Caglioti C., Leuzzi G., Siniscalchi A., De Sarro G., Gallelli L. (2013). Pharmacokinetic drug–drug interaction and their implication in clinical management. J. Res. Med. Sci..

[8] Sánchez-Fidalgo S., Guzmán-Ramos M.I., Galván-Banqueri M., Bernabeu-Wittel M., Santos-Ramos B. (2017). Prevalence of drug interactions in elderly patients with multimorbidity in primary care. Int. J. Clin. Pharm..

[9] Moradi O., Karimzadeh I., Davani-Davari D., Shafiekhani M., Sagheb M.M., Raees-Jalali G.A. (2020). Drug–Drug Interactions among Kidney Transplant Recipients in the Outpatient Setting. Int. J. Organ Transpl. Med..

[10] Hughes J.E., Russo V., Walsh C., Menditto E., Bennett K., Cahir C. (2021). Prevalence and factors associated with potential drug–drug interactions in older community-dwelling adults: A prospective cohort study. Drugs Aging.

[11] Dahri K., Araujo L., Chen S., Bagri H., Walia K., Lau L., Legal M. (2022). Community pharmacist perceptions of drug–drug interactions. Can. Pharm. J..

[12] Hazen A.C., Sloeserwij V.M., Zwart D.L., de Bont A.A., Bouvy M.L., de Gier J.J., de Wit N.J., Leendertse A.J. (2015). Design of the POINT study: Pharmacotherapy Optimisation through Integration of a Non-dispensing pharmacist in a primary care Team (POINT). BMC Fam. Pract..

[13] Eurostat (2024). Luxembourg: European Commission. https://ec.europa.eu/eurostat/statistics-explained/index.php?title=Population_structure_and_ageing.

[14] Barrons R. (2004). Evaluation of personal digital assistant software for drug interactions. Am. J. Health Syst. Pharm..

[15] Jazbar J., Locatelli I., Horvat N., Kos M. (2018). Clinically relevant potential drug–drug interactions among outpatients: A nation-wide database study. Res. Soc. Adm. Pharm..

[16] Toivo T.M., Mikkola J., Laine K., Airaksinen M. (2016). Identifying high risk medications causing potential drug–drug interac-tions in outpatients: A prescription database study based on an online surveillance system. Res. Social Admin. Pharm..

[17] Magro L., Arzenton E., Leone R., Stano M.G., Vezzaro M., Rudolph A., Castagna I., Moretti U. (2021). Identifying and Characterizing Serious Adverse Drug Reactions Associated with Drug–Drug Interactions in a Spontaneous Reporting Database. Front. Pharmacol..

[18] Mirosevic Skvrce N., Macolic Sarinic V., Mucalo I., Krnic D., Bozina N., Tomic S. (2011). Adverse drug reactions caused by drug–drug interactions reported to Croatian Agency for Medicinal Products and Medical Devices: A retrospective observational study. Croat. Med. J..

[19] Létinier L., Cossin S., Mansiaux Y., Arnaud M., Salvo F., Bezin J., Thiessard F., Pariente A. (2019). Risk of Drug–Drug Interactions in Out-Hospital Drug Dispensings in France: Results from the DRUG–Drug Interaction Prevalence Study. Front. Pharmacol..

[20] Annual Report on Drug Utilisation for 2023—Croatian Document. https://halmed.hr/Novosti-i-edukacije/Publikacije-i-izvjesca/Izvjesca-o-potrosnji-lijekova/Izvjesce-o-potrosnji-lijekova-u-Republici-Hrvatskoj-u-2023/.

[21] Patel P.S., Rana D.A., Suthar J.V., Malhotra S.D., Patel V.J. (2014). A study of potential adverse drug–drug interactions among pre-scribed drugs in medicine outpatient department of a tertiary care teaching hospital. J. Basic Clin. Pharm..

[22] Maher R.L., Hanlon J., Hajjar E.R. (2014). Clinical consequences of polypharmacy in elderly. Expert Opin. Drug Saf..

[23] Masnoon N., Shakib S., Kalisch-Ellett L., Caughey G.E. (2017). What is polypharmacy? A systematic review of definitions. BMC Geriatr..

[24] Scotti S., Scotti L., Galimberti F., Xie S., Casula M., Olmastroni E. (2025). Operational definitions of polypharmacy and their association with all-cause hospitalization risk: A conceptual framework using administrative databases. Pharmacy.

[25] Ngcobo N.N. (2025). Influence of ageing on the pharmacodynamics and pharmacokinetics of chronically administered medicines in geriatric patients: A review. Clin. Pharmacokinet..

[26] Hajizadeh A., Hafezi R., Torabi F., Akbari Sari A., Tajvar M. (2025). Consequences of population ageing on health systems: A concep-tual framework for policy and practice. Ethiop. J. Health Sci..

[27] Horvath T., Leoni T., Reschenhofer P., Spielauer M. (2025). The impact of ageing, socio-economic differences and the evolution of morbidity on future health expenditure—A dynamic microsimulation. BMC Health Serv. Res..

[28] Marinovic I., Bacic Vrca V., Samardzic I., Marusic S., Grgurevic I. (2021). Potentially inappropriate medications involved in drug–drug interactions at hospital discharge in Croatia. Int. J. Clin. Pharm..

[29] Kawai T., Momo K. (2024). Impact of potentially inappropriate medications on emergency ambulance admissions in geriatric patients after discharge. Pharmazie.

[30] 2023 American Geriatrics Society Beers Criteria^®^ Update Expert Panel (2023). American Geriatrics Society 2023 updated AGS Beers Criteria^®^ for potentially inappropriate medication use in older adults. J. Am. Geriatr. Soc..

[31] Renom-Guiteras A., Meyer G., Thürmann P.A. (2015). The EU(7)-PIM list: A list of potentially inappropriate medications for older people consented by experts from seven European countries. Eur. J. Clin. Pharmacol..

[32] Somogyi-Végh A., Ludányi Z., Erdős Á., Botz L. (2019). Countrywide prevalence of critical drug interactions in Hungarian outpatients: A retrospective analysis of pharmacy dispensing data. BMC Pharmacol. Toxicol..

